# Fluid-Fluid Interfaces of Multi-Component Mixtures in Local Equilibrium

**DOI:** 10.3390/e20040250

**Published:** 2018-04-04

**Authors:** Dick Bedeaux, Signe Kjelstrup

**Affiliations:** PoreLab, Department of Chemistry, Norwegian University of Science and Technology, 7491 Trondheim, Norway

**Keywords:** Gibbs interface, surface equation of state, dividing interface, local equilibrium, dynamic boundary conditions, phase transition

## Abstract

We derive in a new way that the intensive properties of a fluid-fluid Gibbs interface are independent of the location of the dividing surface. When the system is out of global equilibrium, this finding is not trivial: In a one-component fluid, it can be used to obtain the interface temperature from the surface tension. In other words, the surface equation of state can serve as a thermometer for the liquid-vapor interface in a one-component fluid. In a multi-component fluid, one needs the surface tension and the relative adsorptions to obtain the interface temperature and chemical potentials. A consistent set of thermodynamic properties of multi-component surfaces are presented. They can be used to construct fluid-fluid boundary conditions during transport. These boundary conditions have a bearing on all thermodynamic modeling on transport related to phase transitions.

## 1. Introduction

To understand transport through, into, and along interfaces is of practical importance in physics, chemistry biology, and engineering [1,2,3,4,5,6,7,8]. Large efforts have been made to develop a rigorous and thermodynamically consistent description of surfaces at global equilibrium and away from this condition. Gibbs laid the foundation in his description of surfaces at equilibrium, using excess variables for energy and mass [9]. The method of dynamic boundary conditions [10,11] was proposed in 1976 to deal with surfaces away from equilibrium. The method provides a description of interface transport phenomena that are compatible with the second law of thermodynamics. Current methods for interface transport often do not possess the symmetry required by this method (Onsager symmetry) [3]. In spite of the agreement with symmetry requirements and the second law, the dynamic boundary method has not yet gained common use. One reason may be that there is still only scattered knowledge on the validity of its fundamental assumptions. This article aims to mend this situation by presenting rigorous evidence for the validity and applicability of the basic assumptions of the theory of non-equilibrium thermodynamics for interfaces.

As in the original method [10,11] and later work [3], we shall use Gibbs definition of excess thermodynamic properties [9] to describe the interface. It is well known that these properties depend on a mathematical construct, namely, the dividing surface. The dividing surface has a location that needs to be defined. But can a thermodynamic description depend on a mathematical construct, one may therefore ask? The intensity of reflected light, for instance, certainly does not depend on this location [12], and neither does the surface tension. We shall see that this is also the case for other properly chosen surface excess variables.

In the dynamic boundary method [10,11], the crucial step was to proclaim the surface between two bulk phases to be an autonomous system, also away from global equilibrium. Gibbs was working with interfaces between phases in global equilibrium. In global equilibrium, the temperature and the chemical potentials are everywhere the same and well defined. But can we define such variables in a unique way, when the system is away from global equilibrium and the variables are everywhere different? By proclaiming the surface with its excess variables to be an autonomous system, we imply that the system is in local equilibrium; or that thermodynamic equations apply. The assumption is central in non-equilibrium thermodynamics. The theory builds on the assumption of local equilibrium and of independence of mathematical constructs. Evidence for both properties shall be presented and examined for an interface of multi-component fluids in this article.

Away from global equilibrium, the temperature and chemical potentials of the surface will in general differ from the values of the adjacent bulk phases. A measured surface tension may therefore disagree with the value that can be predicted from the variables of the adjacent homogeneous phase. This problem has lead to the introduction of the concept of a dynamic surface tension [13]. Such a variable may turn out not to be necessary, with more information of the variables of the autonomous surface [14].

The present paper addresses these and related questions. We shall see that a unique definition of surface variables exists, independent of the chosen location of the dividing surface. Furthermore, we shall propose that the property of local equilibrium can be actively used to find surface properties. From knowledge of proper thermodynamic relations for one choice of the location of the dividing surface, one can find the relations for another choice, through transformation of excess densities. This can also help clarify misunderstandings in the literature.

We aim to clarify definitions used in the thermodynamic description of a fluid-fluid interface of mixtures. We restrict ourselves to flat interfaces, but the analysis can be extended to curved ones [10,11]. We consider also only cases where gradients and fluxes are in the direction normal to the surface.

Savin et al. [7] and Öttinger et al. [9] addressed the same problems using the concept of gauge invariance. A gauge transformation is a displacement of the dividing surface over an atomistic distance. The temperature and chemical potentials of the surface were found to be gauge-invariant, whereas the excess densities were gauge-variant. Schweizer et al. [8] verified the results for a one-component liquid-vapor interface, using molecular dynamics simulations. The conclusions of these papers support those discussed here. Nevertheless, we consider it useful to further strengthen the conclusions, following a somewhat different path, as outlined below.

The dividing surface can essentially be displaced in two ways. One can invoke a notional change only. This means that a different dividing surface is chosen without physically moving the original surface. The other way is to physically move the surface to a new location. In the present paper, we are only concerned with notional changes. A notional change is the same as a gauge transformation. This means that the results obtained earlier will be obtained again, but in a different way.

We start by defining in Section 2 what we mean by the dividing surface and an excess density, and show how the concept of excess densities depend on the surface location. Molecular dynamic simulations or density functional theories can be used to obtain continuous profiles of energy- and mass-densities across interfaces. In the interfacial region these profiles change considerably over the thickness of the surface. Gibbs used the profiles to derive his concept of a surface variable. Section 3 presents the Gibbs excess thermodynamic properties of a surface. We shall use the condition of global equilibrium to find an equivalent variable set, a set that is gauge-invariant or does not depend on the location of the surface.

From the set of gauge-invariant excess densities in Section 4, we proceed in Section 5 to show that a corresponding set of surface intensive variables is also gauge-invariant. This knowledge in combination with the assumption of local equilibrium can be actively used to find unknown properties of a surface. We discuss how we can measure thermodynamic quantities of the surface away from global equilibrium. A measurement of, for example, the surface tension can then be regarded as equivalent to a temperature measurement.

The property of local equilibrium has a remarkable consequence; away from global equilibrium, the equation of state for the surface, once the other variables are known, can be used to find dependent variables. Experiments to verify such relations will be proposed.

## 2. Excess Densities Defined

We recapitulate Gibbs’ definition of excess variables and identify the problem with location-dependent thermodynamic properties. The excess density of component *j* in moles per unit of surface area is

(1) $$c_{j}^{\mathrm{exc}}\left(x^{s}\right)\equiv \int \left[c_{j}\left(x\right)-c_{j}^{+}\left(x\right)\theta \left(x-x^{s}\right)-c_{j}^{-}\left(x\right)\theta \left(x^{s}-x\right)\right]dx$$

where the integral is over the length of the system, θ is the Heaviside function (1 for a positive and 0 for a negative argument), *x* is the coordinate measured normal to the surface, and $x^{s}$ is the chosen location of the dividing surface. On a molecular scale, the density $c_{j}\left(x\right)$ changes continuously throughout the interfacial region. The values of $c_{j}^{l}\left(x\right)$ and $c_{j}^{g}\left(x\right)$ are the extrapolated densities of the component *j* in the two fluid phases. There are *n* components. We use the symbol $c_{j}^{\mathrm{exc}}$ to denote the excess number of moles per surface area [15,16,17]. The symbol $\Gamma_{j}$ has been used for the same property.

The variation in the densities of the bulk phases over the thickness of the interfacial region is negligible. All densities depend on the time *t*, but this will not be explicitly indicated. The excess energy and entropy densities are also defined per unit of surface area. The value of $c_{j}^{\mathrm{exc}}$ depends on the choice of the location, $x^{s}$, of the dividing surface. A common and convenient choice of this location is the equimolar surface $x_{j}^{\mathrm{es}}$ of component *j*. This is defined by

(2) $$c_{j}^{\mathrm{exc}}\left(x_{j}^{\mathrm{es}}\right)=0.$$

The location of an equimolar surface refers to one component. When the equimolar surface of one component is used as a dividing surface, the excess densities of all the other components are not equal to zero. Excess molar densities can be both positive and negative.

The surface tension (in Pa·m = J·m${}^{-2}$) is defined by minus the excess parallel pressure and is equal to [5,9]

(3) $$\gamma^{}\left(x^{s}\right)\equiv -p_{∥}^{\mathrm{exc}}\left(x^{s}\right)\equiv -\int \left[p_{∥}\left(x\right)-p_{∥}^{+}\left(x\right)\theta \left(x-x^{s}\right)-p_{∥}^{-}\left(x\right)\theta \left(x^{s}-x\right)\right]dx.$$

The excess internal energy density (in J·m${}^{-2}$) is

(4) $$u^{\mathrm{exc}}\left(x^{s}\right)\equiv \int \left[u\left(x\right)-u^{+}\left(x\right)\theta \left(x-x^{s}\right)-u^{-}\left(x\right)\theta \left(x^{s}-x\right)\right]dx.$$

The excess Helmholtz energy, $f^{\mathrm{exc}}$, the excess entropy, $s^{\mathrm{exc}}$, and the excess enthalpy, $h^{\mathrm{exc}}$, of the surface are given by similar definitions. The excess Gibbs energy is

(5) $$\begin{array}{c} \begin{array}{cc} g^{\mathrm{exc}}\left(x^{s}\right) & \equiv \int [g\left(x\right)-g^{+}\left(x\right)\theta \left(x-x^{s}\right)-g^{-}\left(x\right)\theta \left(x^{s}-x\right)]dx\\ & =\sum_{j=1}^{n}\int \left[c_{j}\left(x\right)\mu_{j}\left(x\right)-c_{j}^{+}\left(x\right)\mu_{j}^{+}\left(x\right)\theta \left(x-x^{s}\right)-c_{j}^{-}\left(x\right)\mu_{j}^{-}\left(x\right)\theta \left(x^{s}-x\right)\right]dx \end{array} \end{array}$$

where $\mu_{j}$ are the chemical potentials.

The extrapolated parallel pressures $p_{∥}^{+}\left(x\right)$ and $p_{∥}^{-}\left(x\right)$ of a flat surface away from the surface are equal to the extrapolated normal pressures $p_{n}^{+}\left(x\right)$ and $p_{n}^{-}\left(x\right)$. The normal pressures are the same and constant in the interfacial region, $p_{n}^{+}\left(x\right)=p_{n}^{-}\left(x\right)=p_{n}$. This is the reason why the surface tension of a flat surface is independent of the location of the dividing surface [5,9]. All excess densities, except the surface tension, depend on the location $x^{s}$ of the dividing surface.

The excess entropy production is standardly found from the Gibbs equation and the balance equations for the excess mass, energy, and entropy densities [3]. The entropy production of the surface is an absolute quantity. It cannot depend on any reference value. The fact that the excess properties depend on the location of the dividing surface is therefore not convenient.

We need to deal with this problem in order to understand the role of the dividing surface, and how it disappears from the entropy production. This will be done in two steps. We introduce a special reference for the chemical potentials in the first step (Section 3). We suppose in the second step that the validity of local equilibrium implies that the wanted invariance is also true. The needed relations can be tested experimentally (Section 4).

## 3. Thermodynamic Properties of Gibbs Surface

### 3.1. The Interface at Global Equilibrium with the Surroundings

The purpose of this subsection is to show that a set of geometry-dependent, excess densities in global equilibrium can be replaced by a set of absolute intensive variables, provided that we introduce a convenient reference chemical potential for the components.

Take the excess functions of the liquid-vapor interface as an example. In order to denote *global equilibrium*, we introduce the subscript eq. For an arbitrary choice of the dividing surface, we have from Gibbs [9]

(6) $$\begin{array}{cc} \gamma_{eq}^{s} & =f_{eq}^{s}-\sum_{j=1}^{n}\mu_{j,eq}c_{j,eq}^{s}\\ u_{eq}^{s} & =f_{eq}^{s}+T_{eq}s_{eq}^{s}=T_{eq}s_{eq}^{s}+\gamma_{eq}^{s}+\sum_{j=1}^{n}\mu_{j,eq}c_{j,eq}^{s}\\ h_{eq}^{s} & =u_{eq}^{s}-\gamma_{eq}^{s}=T_{eq}s_{eq}^{s}+\sum_{j=1}^{n}\mu_{j,eq}c_{j,eq}^{s}\\ g_{eq}^{s} & =h_{eq}^{s}-T_{eq}s_{eq}^{s}=\sum_{j=1}^{n}\mu_{j,eq}c_{j,eq}^{s}. \end{array}$$

Superscript s is used to indicate excess densities of the surface that depend on the location $x^{s}$ of the dividing surface. The excess quantities depend on the temperature and all but one of the chemical potentials. In equilibrium, the temperature and the chemical potentials are constant throughout the system and are therefore accessible experimentally via the bulk phase values. The definitions of Equations (3)–(5) apply.

The thermodynamic excess densities $u_{eq}^{s}$, $f_{eq}^{s}$, $h_{eq}^{s}$, and $g_{eq}^{s}$ as well as $\mu_{1}^{s},\cdots ,\mu_{n-1}^{s}$ need a reference value to be uniquely defined. In equilibrium, one chemical potential depends on the temperature and the other chemical potentials. We then renumber the components so that this component has number *n*. We now use $\mu_{n,eq}c_{eq}^{s}\equiv \mu_{n,eq}\sum_{j=1}^{n}c_{j,eq}^{s}$ to define absolute excess densities:

(7) $$\begin{array}{c} \begin{array}{cc} {\hat{u}}_{eq}^{s} & \equiv u_{eq}^{s}-\mu_{n,eq}c_{eq}^{s},\;\;{\hat{f}}_{eq}^{s}\equiv f_{eq}^{s}-\mu_{n,eq}c_{eq}^{s},\;\;{\hat{h}}_{eq}^{s}\equiv h_{eq}^{s}-\mu_{n,eq}c_{eq}^{s}\\ {\hat{g}}_{eq}^{s} & \equiv g_{eq}^{s}-\mu_{n,eq}c_{eq}^{s}=\sum_{j=1}^{n-1}\left(\mu_{j,eq}-\mu_{n,eq}\right)c_{j,eq}^{s}=\sum_{j=1}^{n-1}\mu_{jn,eq}c_{j,eq}^{s} \end{array} \end{array}$$

where $\mu_{jn,eq}\equiv \mu_{j,eq}-\mu_{n,eq}$ becomes absolute because identical standard chemical potentials can be chosen, and the difference can be found by experiments. Equation (6) can now be alternatively written as

(8) $$\begin{array}{cc} \gamma_{eq}^{s} & ={\hat{f}}_{eq}^{s}-\sum_{j=1}^{n-1}\mu_{jn,eq}c_{j,eq}^{s}\\ {\hat{u}}_{eq}^{s} & ={\hat{f}}_{eq}^{s}+T_{eq}s_{eq}^{s}=T_{eq}s_{eq}^{s}+\gamma_{eq}^{s}+\sum_{j=1}^{n-1}\mu_{jn,eq}c_{j,eq}^{s}\\ {\hat{h}}_{eq}^{s} & ={\hat{u}}_{eq}^{s}-\gamma_{eq}^{s}=T_{eq}s_{eq}^{s}+\sum_{j=1}^{n-1}\mu_{jn,eq}c_{j,eq}^{s}\\ {\hat{g}}_{eq}^{s} & ={\hat{h}}_{eq}^{s}-T_{eq}s_{eq}^{s}=\sum_{j=1}^{n-1}\mu_{jn,eq}c_{j,eq}^{s}. \end{array}$$

These redefined excess energy densities of the surface depend only on $T_{eq},\;\mu_{1n,eq},\cdots ,\mu_{\left(n-1\right)n,eq}$. They are all absolute excess densities.

In global equilibrium, the set $T_{eq},\mu_{1n,eq},\cdots ,\mu_{\left(n-1\right)n,eq}$ is the same throughout the bulk phases and the interface. The values of the homogeneous phases can be found experimentally and applied to the interface. We can then tabulate the set of densities for the homogeneous phases, the set of excess densities for the surface, and $\mu_{n,eq}$ as a function of $T_{eq},\mu_{1n,eq},\cdots ,\mu_{\left(n-1\right)n,eq}$. The tables of the excess densities can be used as “equations of state” for the interface. These lists will be further discussed below.

### 3.2. The Interface Away from Global Equilibrium

We explain here the meaning of local equilibrium for the interface, and find under these circumstances that there is a relation between the densities in and at the interface.

A fluid-fluid interface can be studied also away from global equilibrium. Experimentally we can, for instance, measure the surface tension. From molecular dynamics simulations or with density functional methods, we can obtain density- and other profiles through the surface when it is exposed to gradients. Such profiles have been written as [15,16,17]:

(9) $$\begin{array}{c} \begin{array}{cc} u(x) & =T\left(x\right)s\left(x\right)-p_{∥}\left(x\right)+\sum_{j=1}^{n}\mu_{j}\left(x\right)c_{j}\left(x\right)\\ & =f(x)+T(x)s(x)\\ & =h\left(x\right)-p_{∥}\left(x\right)\\ & =g\left(x\right)+T\left(x\right)s\left(x\right)-p_{∥}\left(x\right) \end{array} \end{array}$$

where $g\left(x\right)=\sum_{j=1}^{n}\mu_{j}\left(x\right)c_{j}\left(x\right)\equiv \sum_{j=1}^{n}g_{j}\left(x\right)$. (We have not explicitly indicated a possible time dependence.) The densities u(x),f(x),h(x),andg(x) as well as the chemical potentials $\mu_{1}\left(x\right),\cdots ,\mu_{n-1}\left(x\right)$ need a reference value. We use the same reference as above and define the absolute densities

(10) $$\begin{array}{c} \begin{array}{cc} \hat{u}\left(x\right) & \equiv u\left(x\right)-\mu_{n}\left(x\right)c\left(x\right)=T\left(x\right)s\left(x\right)-p_{∥}\left(x\right)+\sum_{j=1}^{n-1}\mu_{jn}\left(x\right)c_{j}\left(x\right)\\ & =f\left(x\right)-\mu_{n}\left(x\right)c\left(x\right)+T\left(x\right)s\left(x\right)\equiv \hat{f}\left(x\right)+T\left(x\right)s\left(x\right)\\ & =h\left(x\right)-\mu_{n}\left(x\right)c\left(x\right)-p_{∥}\left(x\right)\equiv \hat{h}\left(x\right)-p_{∥}\left(x\right)\\ & =g\left(x\right)-\mu_{n}\left(x\right)c\left(x\right)+T\left(x\right)s\left(x\right)-p_{∥}\left(x\right)\equiv \hat{g}\left(x\right)+T\left(x\right)s\left(x\right)-p_{∥}\left(x\right) \end{array} \end{array}$$

where $c\left(x\right)\equiv \sum_{j=1}^{n}c_{j}\left(x\right)$ and $\mu_{jn}\left(x\right)\equiv \mu_{j}\left(x\right)-\mu_{n}\left(x\right)$, which are also absolute. Note that $\hat{g}\left(x\right)=\sum_{j=1}^{n-1}\mu_{jn}\left(x\right)c_{j}\left(x\right)$. Following the procedure outlined above, we take the excesses of these expressions and obtain

(11) $$\begin{array}{cc} {\hat{u}}^{\mathrm{exc}} & ={\left(Ts\right)}^{\mathrm{exc}}+{\hat{f}}^{\mathrm{exc}}\\ & =\gamma^{}+{\hat{h}}^{\mathrm{exc}}\\ & ={\left(Ts\right)}^{\mathrm{exc}}+\gamma +{\hat{g}}^{\mathrm{exc}} \end{array}$$

where ${\hat{g}}^{\mathrm{exc}}=\sum_{j=1}^{n-1}{\hat{g}}_{jn}^{\mathrm{exc}}$ and $\gamma \equiv -p_{∥}^{\mathrm{exc}}$. Furthermore, ${\left(Ts\right)}^{\mathrm{exc}}$ and ${\hat{g}}_{jn}^{\mathrm{exc}}={\left(\mu_{jn}c_{j}\right)}^{\mathrm{exc}}$ are defined similarly to the other excess densities. All excess densities with a hat are now absolute.

Local equilibrium means that normal thermodynamic equations apply. For a surface in local equilibrium, all surface excess densities have the equilibrium value given by temperature $T^{s}$ and relative chemical potentials $\mu_{1n}^{s},\cdots ,\mu_{\left(n-1\right)n}^{s}$. For a surface in local equilibrium, we can also convert the above expressions into Gibbs’ relations for the surface, also in the presence of external fields.

In equilibrium, we can identify the temperature and the chemical potentials of the surface:

(12) $$\begin{array}{cc} T^{s} & =\frac{{\left(Ts\right)}^{\mathrm{exc}}}{s^{\mathrm{exc}}}\\ \mu_{jn}^{s} & =\frac{{\hat{g}}_{jn}^{\mathrm{exc}}}{c_{j}^{\mathrm{exc}}}\;\;\;j=1,\cdots ,n-1. \end{array}$$

We furthermore identify

(13) $$\begin{array}{cc} \gamma^{s} & =\gamma ,\;\;\;c^{s}=c^{\mathrm{exc}},\;\;\;{\hat{u}}^{s}={\hat{u}}^{\mathrm{exc}},\;\;\;{\hat{h}}^{s}={\hat{h}}^{\mathrm{exc}}\\ s^{s} & =s^{\mathrm{exc}},\;\;\;{\hat{g}}_{j}^{s}={\hat{g}}_{j}^{\mathrm{exc}},\;\;\;{\hat{f}}^{s}={\hat{f}}^{\mathrm{exc}}. \end{array}$$

By furthermore substituting Equations (12) and (13) into Equation (11), we obtain

(14) $$\begin{array}{cc} u^{s} & =f^{s}+T^{s}s^{s}=h^{s}+\gamma^{s}\\ & =T^{s}s^{s}+\gamma^{s}+g^{s}=T^{s}s^{s}+\gamma^{s}+\sum_{j=1}^{n}\mu_{j}^{s}c_{j}^{s}. \end{array}$$

For the absolute excess densities, the equivalent equations are

(15) $$\begin{array}{cc} {\hat{u}}^{s} & ={\hat{f}}^{s}+T^{s}s^{s}={\hat{h}}^{s}+\gamma^{s}\\ & =T^{s}s^{s}+\gamma^{s}+{\hat{g}}^{s}=T^{s}s^{s}+\gamma^{s}+\sum_{j=1}^{n-1}\mu_{jn}^{s}c_{j}^{s}. \end{array}$$

These are the relations given originally for autonomous interfaces by Gibbs, cf. Equation (8). While Gibbs gave these relations only for a fluid-fluid interface in global equilibrium, we have now shown, through the use of the identifications of Equation (12), that they are also valid away from equilibrium.

The importance of this cannot be understated. It means that the theory of non-equilibrium thermodynamics can be used to describe transport along, into, and through the surface (see [3]). From the Gibbs relation and the balance equations for energy, entropy, and masses, we can obtain an expression for the entropy production away from global equilibrium. Relations can also be formulated for surface excess density fluctuations [11]. The entropy production can be used to identify relevant thermodynamic forces and fluxes, and their linear relations leads to a complete description of the time dependence of the properties of the surface.

Two important questions remain, however. How can we determine the temperature $T^{s}$ and the relative chemical potentials $\mu_{1n}^{s},\cdots ,\mu_{\left(n-1\right)n}^{s}$ of a surface in a non-equilibrium system? Moreover, why are $T^{s}$, $\mu_{1n}^{s},\cdots ,\mu_{\left(n-1\right)n}^{s}$ independent of the choice of the location of the dividing surface (gauge-invariant)?

## 4. Excess Densities and the Choice of Dividing Surface

To answer the questions formulated in the last section, consider for the sake of argument the two most common choices for the dividing surface: the equimolar surfaces and the surface of tension. The position, $x_{j}^{\mathrm{es}}$, of the equimolar surface for component *j* is defined by

(16) $$0=\int [c_{j}\left(x\right)-c_{j}^{+}\left(x\right)\theta \left(x-x_{j}^{\mathrm{es}}\right)-c_{j}^{-}\left(x\right)\theta \left(x_{j}^{\mathrm{es}}-x\right)]dx$$

where the integral is over the whole box, cf. Equations (1) and (2). The position, $x^{\mathrm{st}}$, of the surface of tension, on the other hand, is defined by setting the first moment of the excess parallel pressure equal to zero

(17) $$0=\int \left(x-x^{\mathrm{st}}\right)\left[p_{∥}\left(x\right)-p_{∥}^{+}\left(x\right)\theta \left(x-x^{\mathrm{st}}\right)-p_{∥}^{-}\left(x\right)\theta \left(x^{\mathrm{st}}-x\right)\right]dx.$$

Other locations are possible. One can, for instance, set the excess internal energy density equal to zero and use the equation to determine the position of the corresponding dividing surface.

For the surface of tension, all excess molar densities are not equal to zero. For the equimolar surfaces, the first moment of the excess parallel pressure is not equal to zero. With knowledge of excess densities for a particular choice of the dividing surface, one can find the set of corresponding excess densities for other choices of the dividing surface. Consider the two choices of the location of the dividing surface $x_{1}^{s}$ and $x_{2}^{s}$. We take $x_{1}^{s}<x_{2}^{s}$. Using the definition in Equation (3), we find

(18) $$\begin{array}{cc} {\hat{u}}^{s}\left(x_{2}^{s}\right) & ={\hat{u}}^{s}\left(x_{1}^{s}\right)+\int_{x_{1}^{s}}^{x_{2}^{s}}({\hat{u}}^{+}\left(x\right)-{\hat{u}}^{-}\left(x\right))dx\\ & ={\hat{u}}^{s}\left(x_{1}^{s}\right)+\left(x_{2}^{s}-x_{1}^{s}\right)({\hat{u}}^{+}\left(x_{1}^{s}\right)-{\hat{u}}^{-}\left(x_{1}^{s}\right))\\ & +{\left.\frac{1}{2}{\left(x_{2}^{s}-x_{1}^{s}\right)}^{2}\frac{d}{dx}({\hat{u}}^{+}\left(x\right)-{\hat{u}}^{-}\left(x\right))\right|}_{x=x_{1}^{s}}+\cdots \cdots \end{array}$$

The variation of the extrapolated values of the homogeneous phases, ${\hat{u}}^{+}\left(x\right)$ and ${\hat{u}}^{-}\left(x\right)$ over the thickness of the surface, is negligible. This implies that ${\left|\left(x_{2}^{s}-x_{1}^{s}\right)d\left({\hat{u}}^{+}\left(x\right)-{\hat{u}}^{-}\left(x\right)\right)/dx\right|}_{x=x_{1}^{s}}\ll \left|{\hat{u}}^{+}\left(x_{1}^{s}\right)-{\hat{u}}^{-}\left(x_{1}^{s}\right)\right|$. For this reason, $\left({\hat{u}}^{+}-{\hat{u}}^{-}\right)$ can be taken to be independent of the location of the dividing surface, and Equation (18) gives

(19) $${\hat{u}}^{s}\left(x_{2}^{s}\right)={\hat{u}}^{s1}\left(x_{1}^{s}\right)+\left(x_{2}^{s}-x_{1}^{s}\right)({\hat{u}}^{+}\left(x_{1}^{s}\right)-{\hat{u}}^{-}\left(x_{1}^{s}\right)).$$

Similar expressions are valid for the other excess densities. This then results in

(20a) $$c_{j}^{s}\left(x_{2}^{s}\right)=c_{j}^{s}\left(x_{1}^{s}\right)+\left(x_{2}^{s}-x_{1}^{s}\right)\left(c_{j}^{+}-c_{j}^{-}\right)$$

(20b) $${\hat{u}}^{s}\left(x_{2}^{s}\right)={\hat{u}}^{s}\left(x_{1}^{s}\right)+\left(x_{2}^{s}-x_{1}^{s}\right)\left({\hat{u}}^{+}-{\hat{u}}^{-}\right)$$

(20c) $${\hat{h}}^{s}\left(x_{2}^{s}\right)={\hat{h}}^{s}\left(x_{1}^{s}\right)+\left(x_{2}^{s}-x_{1}^{s}\right)\left({\hat{h}}^{+}-{\hat{h}}^{-}\right)$$

(20d) $${\hat{f}}^{s}\left(x_{2}^{s}\right)={\hat{f}}^{s}\left(x_{1}^{s}\right)+\left(x_{2}^{s}-x_{1}^{s}\right)\left({\hat{f}}^{+}-{\hat{f}}^{-}\right)$$

(20e) $$s^{s}\left(x_{2}^{s}\right)=s^{s}\left(x_{1}^{s}\right)+\left(x_{2}^{s}-x_{1}^{s}\right)\left(s^{+}-s^{-}\right)$$

(20f) $${\hat{g}}_{j}^{s}\left(x_{2}^{s}\right)={\hat{g}}_{j}^{s}\left(x_{1}^{s}\right)+\left(x_{2}^{s}-x_{1}^{s}\right)\left({\hat{g}}_{j}^{+}-{\hat{g}}_{j}^{-}\right)$$

(20g) $$={\hat{g}}_{j}^{s}\left(x_{1}^{s}\right)+\left(x_{2}^{s}-x_{1}^{s}\right)\left(c_{j}^{+}\mu_{jn}^{+}-c_{j}^{-}\mu_{jn}^{-}\right).$$

All relations in Equation (20a) were found to apply in very good approximation for a one-component fluid [18] and for a two-component fluid mixture [16].

Following Gibbs, we introduce the excess concentrations of component *j* relative to component *n*:

(21) $$c_{jn}^{s}\left(x^{s}\right)\equiv c_{j}^{s}\left(x^{s}\right)-c_{n}^{s}\left(x^{s}\right)\frac{c_{j}^{+}-c_{j}^{-}}{c_{n}^{+}-c_{n}^{-}}.$$

It follows from Equation (20a) that

(22) $$c_{jn}^{s}\left(x_{1}^{s}\right)=c_{jn}^{s}\left(x_{2}^{s}\right),\;\;\;j=1,\cdots ,n-1.$$

The relative excess concentrations of a flat surface therefore do not depend on the location of the dividing surface (they are gauge-invariant). This is precisely the reason why Gibbs [9] introduced the relative excess concentrations in global equilibrium.

We can similarly define all other excess densities relative to component *n* by

(23) $$\begin{array}{cc} {\hat{u}}_{\left(n\right)}^{s}\left(x^{s}\right) & ={\hat{u}}^{s}\left(x^{s}\right)-c_{n}^{s}\left(x^{s}\right)\frac{{\hat{u}}^{+}-{\hat{u}}^{-}}{c_{n}^{+}-c_{n}^{-}}\\ {\hat{h}}_{\left(n\right)}^{s}\left(x^{s}\right) & ={\hat{h}}^{s}\left(x^{s}\right)-c_{n}^{s}\left(x^{s}\right)\frac{{\hat{h}}^{+}-{\hat{h}}^{-}}{c_{n}^{+}-c_{n}^{-}}\\ {\hat{f}}_{\left(n\right)}^{s}\left(x^{s}\right) & ={\hat{f}}^{s}\left(x^{s}\right)-c_{n}^{s}\left(x^{s}\right)\frac{{\hat{f}}^{+}-{\hat{f}}^{-}}{c_{n}^{+}-c_{n}^{-}}\\ s_{\left(n\right)}^{s}\left(x^{s}\right) & =s^{s}\left(x^{s}\right)-c_{n}^{s}\left(x^{s}\right)\frac{s^{+}-s^{-}}{c_{n}^{+}-c_{n}^{-}}\\ {\hat{g}}_{jn}^{s}\left(x^{s}\right) & ={\hat{g}}_{j}^{s}\left(x^{s}\right)-c_{n}^{s}\left(x^{s}\right)\frac{{\hat{g}}_{j}^{+}-{\hat{g}}_{j}^{-}}{c_{n}^{+}-c_{n}^{-}}\\ & ={\hat{g}}_{j}^{s}\left(x^{s}\right)-c_{n}^{s}\left(x^{s}\right)\frac{c_{j}^{+}\mu_{jn}^{+}-c_{j}^{-}\mu_{jn}^{-}}{c_{n}^{+}-c_{n}^{-}}. \end{array}$$

All the excess densities relative to component *n* are gauge-invariant or independent of the location of the dividing surface:

(24) $$\begin{array}{c} \begin{array}{cc} {\hat{u}}_{\left(n\right)}^{s}\left(x_{1}^{s}\right) & ={\hat{u}}^{s}\left(x_{2}^{s}\right),\;\;{\hat{h}}_{\left(n\right)}^{s}\left(x_{1}^{s}\right)={\hat{h}}^{s}\left(x_{2}^{s}\right),\;\;{\hat{f}}_{\left(n\right)}^{s}\left(x_{1}^{s}\right)={\hat{f}}^{s}\left(x_{2}^{s}\right)\\ s_{\left(n\right)}^{s}\left(x_{1}^{s}\right) & =s^{s}\left(x_{2}^{s}\right),\;\;{\hat{g}}_{jn}^{s}\left(x_{1}^{s}\right)={\hat{g}}_{j}^{s}\left(x_{2}^{s}\right). \end{array} \end{array}$$

We have shown that the excess densities relative to component *n* do not depend on the location of the dividing surface. This is true in global as well as local equilibrium. Non-equilibrium molecular dynamic (NEMD) simulations [18,19,20] and the square gradient analysis [18] of the liquid-vapor interface for a one-component fluid have confirmed this property. For a two-component fluid mixture, this was also documented [16] using the square gradient analysis.

## 5. The Autonomous Surface

### 5.1. Invariance to Choice of Surface Location (Gauge Invariance)

In local equilibrium, all excess surface densities have their equilibrium values at the surface temperature $T^{s}$ and chemical potential differences $\mu_{1n}^{s},\cdots ,\mu_{\left(n-1\right)n}^{s}$:

(25) $$\begin{array}{cc} \gamma & \equiv \gamma_{eq}\left(T^{s},\mu_{1n}^{s},\ldots ,\mu_{\left(n-1\right)n}^{s}\right),\;\;\;c_{jn}^{s}\equiv c_{jn,eq}^{s}\left(T^{s},\mu_{1n}^{s},\ldots ,\mu_{\left(n-1\right)n}^{s}\right)\\ {\hat{u}}^{s} & \equiv {\hat{u}}_{eq}^{s}\left(T^{s},\mu_{1n}^{s},\ldots ,\mu_{\left(n-1\right)n}^{s}\right),\;\;\;{\hat{h}}^{s}\equiv {\hat{h}}_{eq}^{s}\left(T^{s},\mu_{1n}^{s},\ldots ,\mu_{\left(n-1\right)n}^{s}\right)\\ s^{s} & \equiv s_{eq}^{s}\left(T^{s},\mu_{1n}^{s},\ldots ,\mu_{\left(n-1\right)n}^{s}\right),\;\;\;{\hat{f}}^{s}\equiv {\hat{f}}_{eq}^{s}\left(T^{s},\mu_{1n}^{s},\ldots ,\mu_{\left(n-1\right)n}^{s}\right), \end{array}$$

and the same is true for ${\hat{u}}_{\left(n\right)}^{s}$, ${\hat{h}}_{\left(n\right)}^{s}$, $s_{\left(n\right)}^{s}$ and ${\hat{f}}_{\left(n\right)}^{s}$. It follows that the temperature and the chemical potential differences are also independent of the location of the dividing surface (gauge-invariant). To see this, take the surface tension and the relative excess concentrations as examples. They are all independent of the location of the dividing surface (Equation (22)) or gauge-invariant.

We can now use knowledge of the equilibrium properties to obtain information of the surface state in local equilibrium (away from global equilibrium). We use the easily obtained properties of global equilibrium to tabulate corresponding values of $\gamma_{eq}\left(T_{eq},\mu_{1n,eq},\cdots ,\mu_{\left(n-1\right)n,eq}\right)$ and $c_{jn,eq}^{s}\left(T_{eq},\mu_{1n,eq},\cdots ,\mu_{\left(n-1\right)n,eq}\right)$ as functions of temperature and chemical potential differences.

Away from global equilibrium, we can next use (thanks to local equilibrium) measured values of the surface tension and relative excess concentrations, and find $T^{s},\mu_{1n}^{s},\cdots ,\mu_{\left(n-1\right)n}^{s}$ based on the table. Since γ and $c_{jn}^{s}$ are gauge-invariant, we will obtain the same $T^{s},\mu_{1n}^{s},\cdots ,\mu_{\left(n-1\right)n}^{s}$, independent of the choice we made for location of the dividing surface. The same applies to $\mu_{n}^{s}$. The only condition used is that of local equilibrium.

The conclusions follow therefore directly from the assumption of local equilibrium. The surface is, in other words, an autonomous system. The validity of local equilibrium or this autonomy must of course be investigated. This has been done using several tools; density functional theory (the square gradient model) for one- and two-component fluids [15,17] and molecular dynamics simulations [18,19,20,21,22,23], cf., the discussion below. It remains a challenge to verify the assumption by experiments.

From the autonomous nature of the surface, it follows that all linear expansion coefficients in Equation (20a) have their equilibrium value at the temperature of the surface. All excess densities of the equilibrium surface are functions of temperature and chemical potential differences alone. The state of the surface is therefore completely given in terms of the $T_{eq},\mu_{1n,eq},\cdots ,\mu_{\left(n-1\right)n,eq}$ alone. By applying Equation (20a) to the equilibrium surface, we find

(26) $$\begin{array}{cc} & c_{j,eq}^{s}\left(T_{eq},\mu_{1n,eq},\cdots ,\mu_{\left(n-1\right)n,eq};x_{2}^{s}\right)-c_{j,eq}^{s}\left(T_{eq},\mu_{1n,eq},\cdots ,\mu_{\left(n-1\right)n,eq};x_{1}^{s}\right)\\ = & \left(x_{2}^{s}-x_{1}^{s}\right)\left[c_{j,eq}^{+}\left(T_{eq},\mu_{1n,eq},\cdots ,\mu_{\left(n-1\right)n,eq}\right)-c_{j,eq}^{-}\left(T_{eq},\mu_{1n,eq},\cdots ,\mu_{\left(n-1\right)n,eq}\right)\right]\\ & {\hat{u}}_{eq}^{s}\left(T_{eq},\mu_{1n,eq},\cdots ,\mu_{\left(n-1\right)n,eq};x_{2}^{s}\right)-{\hat{u}}_{eq}^{\mathrm{es}}\left(T_{eq},\mu_{1n,eq},\cdots ,\mu_{\left(n-1\right)n,eq};x_{1}^{s}\right)\\ = & \left(x_{2}^{s}-x_{1}^{s}\right)\left[{\hat{u}}_{eq}^{+}\left(T_{eq},\mu_{1n,eq},\cdots ,\mu_{\left(n-1\right)n,eq}\right)-{\hat{u}}_{eq}^{-}\left(T_{eq},\mu_{1n,eq},\cdots ,\mu_{\left(n-1\right)n,eq}\right)\right], \end{array}$$

and similar expressions for ${\hat{h}}^{s},s^{s},$ ${\hat{f}}^{s}$, and ${\hat{g}}_{j}^{s}$.

Even when the system is not in global equilibrium, the property of local equilibrium implies that the relations in Equation (26) remain valid, but now at the temperature and n−1 chemical potentials of the surface. This means for a surface in local equilibrium that the extrapolated bulk densities satisfy

(27) $$\begin{array}{cc} c_{j}^{+}-c_{j}^{-} & =c_{j,eq}^{+}\left(T^{s},\mu_{1n}^{s},\cdots ,\mu_{\left(n-1\right)n}^{s}\right)-c_{j,eq}^{-}\left(T^{s},\mu_{1n}^{s},\cdots ,\mu_{\left(n-1\right)n}^{s}\right)\\ {\hat{u}}^{+}-{\hat{u}}^{-} & ={\hat{u}}_{eq}^{+}\left(T^{s},\mu_{1n}^{s},\cdots ,\mu_{\left(n-1\right)n}^{s}\right)-{\hat{u}}_{eq}^{-}\left(T^{s},\mu_{1n}^{s},\cdots ,\mu_{\left(n-1\right)n}^{s}\right), \end{array}$$

and similar expressions for ${\hat{h}}^{s},s^{s},$ ${\hat{f}}^{s}$, and ${\hat{g}}_{j}^{s}$. Equation (27) is equivalent to the statement that the linear coefficients in the expansion in Equation (20a) have their equilibrium values. *After measuring* $T^{s},\mu_{1n}^{s},\cdots ,\mu_{\left(n-1\right)n}^{s}$, the surface tension and the excess molar density differences, Equation (27) can be used to give the density difference in the non-equilibrium state in terms of the tabulated equilibrium density difference.

Equation (27) has been verified with a square gradient model both for the one-component and for the two-component fluid [16,18]. This remains to be proven experimentally. This is an experimental challenge.

### 5.2. The Gibbs, Gibbs-Duhem, and Clapeyron Equations

The Gibbs relation for the surface and the bulk phases in equilibrium is

(28) $$du_{eq}^{\alpha }=T_{eq}ds_{eq}^{\alpha }+\sum_{j=1}^{n}\mu_{j,eq}dc_{j,eq}^{\alpha }$$

where α=s, +, −. Using the absolute internal energy density this can be written as

(29) $$d{\hat{u}}_{eq}^{\alpha }=T_{eq}ds_{eq}^{\alpha }+\sum_{j=1}^{n-1}\mu_{jn,eq}dc_{j,eq}^{\alpha }-c_{eq}^{\alpha }d\mu_{n,eq}.$$

Using the Euler relations of Equations (14) and (15), one obtains the Gibbs-Duhem relations:

(30) $$s_{eq}^{\alpha }dT_{eq}-dp_{eq}^{\alpha }+\sum_{j=1}^{n}c_{j,eq}^{\alpha }d\mu_{j,eq}=0$$

or alternatively

(31) $$s_{eq}^{\alpha }dT_{eq}-dp_{eq}^{\alpha }+\sum_{j=1}^{n-1}c_{j,eq}^{\alpha }d\mu_{jn,eq}+c_{eq}^{\alpha }d\mu_{n,eq}=0$$

where $p_{eq}^{s}=-\gamma_{eq}$.

From Equation (30), one obtains the Clapeyron equations

(32) $$\begin{array}{c} \begin{array}{cc} {\left(\frac{\partial \mu_{j,eq}}{\partial T_{eq}}\right)}_{\mu_{k\neq j,eq}} & =\frac{s_{eq}^{+}-s_{eq}^{-}}{c_{j,eq}^{+}-c_{j,eq}^{-}}\equiv \frac{\Delta s_{eq}}{\Delta c_{j,eq}}\\ {\left(\frac{\partial \mu_{n,eq}}{\partial \mu_{j,eq}}\right)}_{T_{eq},\mu_{k\neq j,n,eq}} & =\frac{c_{j,eq}^{+}-c_{j,eq}^{-}}{c_{n,eq}^{+}-c_{n,eq}^{-}}\equiv \frac{\Delta c_{j,eq}}{\Delta c_{n,eq}}. \end{array} \end{array}$$

Local equilibrium implies that Equations (28)–(32) remain valid locally also when the system is not in global equilibrium. For Equations (28)–(31), this implies that the subscript eq can be taken away while the temperature and the chemical potentials should have a superscript α. For the Clapeyron equations, the condition implies that

(33) $$\begin{array}{c} \begin{array}{cc} {\left(\frac{\partial \mu_{j}^{s}}{\partial T^{s}}\right)}_{\mu_{k\neq j}^{s}} & =\frac{s^{+}-s^{-}}{c_{j}^{+}-c_{j}^{-}}=\frac{\Delta s_{eq}\left(T^{s},\mu_{1n}^{s},\cdots ,\mu_{\left(n-1\right)n}^{s}\right)}{\Delta c_{j,eq}\left(T^{s},\mu_{1n}^{s},\cdots ,\mu_{\left(n-1\right)n}^{s}\right)}\\ {\left(\frac{\partial \mu_{n}^{s}}{\partial \mu_{j}^{s}}\right)}_{T^{s},\mu_{k\neq j,n}^{s}} & =\frac{c_{j}^{+}-c_{j}^{-}}{c_{n}^{+}-c_{n}^{-}}=\frac{\Delta c_{j,eq}\left(T^{s},\mu_{1n}^{s},\cdots ,\mu_{\left(n-1\right)n}^{s}\right)}{\Delta c_{n,eq}\left(T^{s},\mu_{1n}^{s},\cdots ,\mu_{\left(n-1\right)n}^{s}\right)} \end{array} \end{array}$$

where we used Equation (27).

## 6. Discussion and Conclusions

### 6.1. Numerical Evidence for an Autonomous Interface

The surface tension of a one-component fluid-fluid interface (liquid-vapor) was measured as a function of temperature. Johannessen et al. [18] analyzed the properties of the liquid-vapor interface with the square gradient model for a one-component fluid for very large evaporation or condensation velocities (between 0.27 and 1.6 m per second in the vapor phase), and extremely large temperature gradients (like 10${}^{8}$ K/m).

Three temperatures were calculated:

1. $T^{\mathrm{es}}$, the surface temperature from Equation (12) for the equimolar surface;
2. $T^{\mathrm{st}}$, the surface temperature from Equation (12) for the surface of tension;
3. $T_{\gamma }^{s}$, the surface temperature found from the surface tension γ.

The three surface temperatures were in very good approximation the same [18]. This gives us reason to believe that $T_{\gamma }^{s},$ $T^{\mathrm{es}}$ and $T^{\mathrm{st}}$ in fact all are the surface temperature $T^{s}$.

The surface in the computations was clearly in local equilibrium. The surface away from global equilibrium is therefore *autonomous*. The equilibrium as well as the non-equilibrium van der Waals square gradient model are not autonomous in their continuous description of the interfacial region. Nevertheless, the Gibbs surface defined by the excess densities is autonomous. This fact does not depend on the choice of the location of the dividing surface. The property of local equilibrium is therefore true for an arbitrary dividing surface.

For a two-component fluid, two variables are needed to describe the interface. A good choice is the surface tension, γ, and the excess concentration of Component 1 relative to Component 2, $c_{12}^{s}$. This will then serve as a surface temperature and chemical potential gauge. Glavatskiy et al. [16] analyzed the properties of the liquid-vapor interface with the square gradient model for a two-component fluid and established that the surface was described by excess energy and that mass density was in local equilibrium.

### 6.2. Surface Properties and Surface Jumps

Badam et al. [6] measured temperature differences across the surface up to 14.04 K for a water-water-vapor interface. Given that the surface thickness is in the order of a few nanometers, this temperature difference across the surface is substantial. The surface temperature was closer to the extrapolated temperature of the liquid than to the extrapolated temperature of the vapor in the NEMD results by Røsjorde et al. [19,20,21]. Based on the accuracy of the determination of the surface temperature with the van der Waals square gradient model, one can conclude that it is not correct to suppose that the surface temperature is equal to the extrapolated liquid temperature [16,18].

### 6.3. Conclusions

The overall conclusion is that a consistent thermodynamic description of a non-equilibrium interface can be found. When the interface is in local equilibrium, its properties satisfy all the properties formulated by Gibbs for the equilibrium interface. This can then be used in non-equilibrium thermodynamics. Away from global equilibrium, the interface has its own temperature and chemical potentials different from the extrapolated values in the bulk phases.

In a one-component fluid, the temperature away from equilibrium can be obtained from the surface tension from tabulated equilibrium values of the temperature as a function of the surface tension. Once the temperature of the surface is known away from global equilibrium, the theory predicts the non-equilibrium density difference across the surface [18]. It is still an experimental challenge to verify this prediction. To find the surface temperature and chemical potentials of a multi-component fluid away from global equilibrium, the surface tension and the relative excess molar densities are needed [16].
